# Prevalencia mundial de la diabetes mellitus tipo 2 y su relación con el índice de desarrollo humano

**DOI:** 10.26633/RPSP.2017.103

**Published:** 2017-11-30

**Authors:** Miguel Ángel Mendoza Romo, Aldanely Padrón Salas, Patricia Elizabeth Cossío Torres, Manuel Soria Orozco

**Affiliations:** 1 Instituto Mexicano del Seguro Social Instituto Mexicano del Seguro Social San Luis Potosí México Instituto Mexicano del Seguro Social, San Luis Potosí, México.; 2 Departamento de Salud Pública, Facultad de Medicina Departamento de Salud Pública, Facultad de Medicina, Universidad Autónoma de San Luis Potosí San Luis Potosí México Departamento de Salud Pública, Facultad de Medicina, Universidad Autónoma de San Luis Potosí, San Luis Potosí, México.; 3 Facultad de Medicina Universidad Autónoma de San Luis Potosí, San Luis Potosí, México San Luis Potosí México Facultad de Medicina, Universidad Autónoma de San Luis Potosí, San Luis Potosí, México.

**Keywords:** Diabetes mellitus tipo 2, índice de desarrollo humano, Programa de las Naciones Unidas para el Desarrollo, Diabetes mellitus, type 2, human development, United Nations Development Program, Diabetes mellitus tipo 2, desenvolvimento humano, Programa das Nações Unidas para o Desenvolvimento

## Abstract

**Objetivo.:**

*Evaluar la relación entre la prevalencia de diabetes mellitus tipo 2 (DM2) y el índice de desarrollo humano (IDH) por región del mundo en el período 2010–2015*.

**Método.:**

*Se utilizaron los datos de la Federación Internacional de Diabetes para la prevalencia de DM2 (2010–2015) y el IDH del Programa de las Naciones Unidas para el Desarrollo. Se analizaron correlaciones lineales de Spearman entre el IDH y la prevalencia de DM2 y se hicieron regresiones lineales para estimar la relación entre ambos*.

**Resultados.:**

*Se observó que a menor IDH menores son las prevalencias de DM2, y a mayor IDH, mayores son las prevalencias de DM2. A nivel mundial, el IDH explica 8,6% de la varianza de la prevalencia de DM2 (P < 0,0001) y que este comportamiento fue diferente en cada región del mundo*.

**Conclusiones.:**

*El IDH puede influir en la prevalencia de DM2, aunque la relación depende de cada país, región y año analizado*.

## Abstract (en)

**Objective.:**

To evaluate the relationship between the prevalence of type 2 diabetes mellitus (DM2) and the Human Development Index (HDI), by region of the world in the period 2010—2015.

**Method.:**

International Diabetes Federation data were used for DM2 prevalence (2010—2015), together with HDI data (United Nations Development Program). Spearman linear correlations between HDI data and DM2 prevalence were analyzed, and linear regressions were done to estimate the relationship between the two.

**Results.:**

It was observed that lower HDI scores corresponded to lower DM2 prevalence rates, and higher HDI scores to higher DM2 prevalence. At the global level, the HDI explains the 8.6% variance of DM2 prevalence (P < 0.0001) and shows that the situation was different in each region of the world.

**Conclusions.:**

While HDI score may be associated with DM2 prevalence, the relationship between them differs from region to region and from country to country, and depends on the particular year analyzed.

## RESUMO

**Objetivo.:**

Avaliar a relação entre a prevalência de diabetes mellitus tipo 2 (DM2) e o índice de desenvolvimento humano (IDH) por região do mundo no período de 2010 a 2015.

**Métodos.:**

Foram utilizados dados da Federação Internacional de Diabetes para a prevalência da DM2 (2010–2015) e o IDH do Programa das Nações Unidas para o Desenvolvimento. Foram analisadas as correlações lineares de Spearman entre o IDH e a prevalência de DM2 e feitas regressões lineares para estimar a relação entre ambos.

**Resultados.:**

Observou-se que quanto mais baixo o IDH, menores são as prevalências de DM2, e quanto mais alto o IDH, maiores são prevalências de DM2. Ao nível mundial, o IDH explica 8,6% da variança da prevalência de DM2 (P < 0,0001) e este comportamento difere em cada região do mundo.

**Conclusões.:**

O IDH pode influir na prevalência de DM2, embora a relação dependa de cada país, região e ano analisados.

Los grandes cambios sociales y económicos han modificado la morbilidad y mortalidad de los países y explican que ahora afronten el aumento de la prevalencia de enfermedades crónicas como la diabetes mellitus tipo 2 (DM2) (1). La DM2 es una enfermedad crónica multifactorial,que discapacita y mata a un gran porcentaje de la población a nivel mundial (2).

La DM2 empobrece a las personas y a sus familias, y a ellas y a los sistemas de salud de los países les impone una enorme carga económica (3). Los gastos totales anuales de esta enfermedad oscilan entre $US 141,6 millones y 174 mil millones, y se estima que las personas con DM2 gastan al menos el doble de dinero en salud que quienes no la padecen (4, 5).

 Anteriormente, la DM2 se consideraba una enfermedad de ricos y ancianos (6). En cambio, hoy en día se ha arraigado en los países en desarrollo, puesto que en los últimos años más de 80% de las muertes causadas por esta enfermedad se han registrado en países de ingresos bajos y medios y se calcula que su carga de morbilidad aumentará en todo el mundo y en particular en países en desarrollo (7–9).

 En la actualidad, la prevalencia mundial de la DM2 en personas mayores de 18 años ha aumentado de 4,7% (108 millones de personas) en 1980 a 8,5% (422 millones de personas) en 2014 y este aumento ha sido más rápido en los países de ingresos medianos y bajos (8).

Al investigar las causas del aumento de la prevalencia de la DM2 y de otras enfermedades crónicas como la tuberculosis, la infección por el virus de la inmunodeficiencia humana, el asma, la depresión o el cáncer, se ha comprobado que la contribución de los factores biológicos y genéticos no es suficiente para explicarlo (5) y que, en cambio, se han encontrado asociaciones con determinantes sociales, como el nivel socioeconómico, los ingresos, la educación, así como con el índice de desarrollo humano (IDH) (10).

En este sentido, se ha observado que a menor ingreso y educación el riesgo de desarrollar DM2 es de 2 a 4 veces más alto que en las personas con ingresos y educación más altos (5, 11). Además, la pobreza se ha asociado con una esperanza de vida más corta y un aumento de la mortalidad, sobre todo la relacionada con enfermedades crónicas como la DM2 (11–14). Por otro lado, diversos estudios sugieren que el nivel educativo es clave para adoptar comportamientos relacionados con la salud, como la nutrición adecuada y la adopción de estilos de vida saludables y, por lo tanto, que es posible que el nivel educativo actúe como causa fundamental de la enfermedad mediante la utilización de recursos como el conocimiento, porque influye en la capacidad de las personas para reducir los riesgos, prevenir o retrasar la aparición de la DM2 (15–20). Por su parte, un IDH bajo se ha asociado con un aumento de la incidencia de mortalidad de enfermedades crónicas, lo que refleja la presencia de desigualdades en los factores de riesgo como el acceso, la calidad, la infraestructura y la cobertura de los servicios de salud (21–23).

Teniendo en cuenta lo anterior, cuando se trata de explicar las condiciones en que viven todos los individuos de un país y cómo éstas se relacionan con la prevalencia de una enfermedad, como la DM2, el IDH, por su composición, se puede considerar como primer indicador a nivel macro de los determinantes sociales de la salud (24).

 El IDH se utiliza como una propuesta de aproximación multifacética que permite comparar el desarrollo entre países del mundo. Este índice consta de tres componentes: i) educación, considerado como un importante elemento que ayuda a la población a tener más oportunidades de empleo y desarrollo profesional, ii) salud, representado por la esperanza de vida al nacer, y iii) el producto interior bruto (PIB), representado por el ingreso per cápita (25). Un IDH alto indica que los habitantes de un país tienen una vida larga y saludable, acceso a educación y un estándar de vida satisfactorio, lo cual se reflejaría en un país con baja morbilidad por DM2. No obstante, una mejora de la economía o de los bienes materiales puede no traducirse en valores humanos, buena calidad de vida o bienestar en personas con alguna enfermedad crónica como la diabetes.

Para corroborar lo anterior, el objetivo de este estudio fue evaluar la relación de la prevalencia de la DM2 y el IDH por región del mundo de acuerdo con la información proporcionada por la Federación Internacional de Diabetes (FID) y por país, a fin de conocer su comportamiento a nivel global en el periodo comprendido entre 2010 y 2015.

## MATERIALES Y MÉTODOS

Se realizó un estudio ecológico con información de la prevalencia de la DM2 y del IDH del período comprendido entre 2010 y 2015 de los países de todo el mundo, conglomerados por regiones según la FID (África, América Central y del Sur, América del Norte y el Caribe, Oriente medio y Norte de África, Europa, Pacífico Occidental y Sudeste asiático).

La información de la prevalencia de DM2 se obtuvo de los atlas de la FID publicados para los años 2010, 2011, 2012,2013 y 2015 (26–30). Para 2014, como no había datos publicados, se calcularon los valores a partir de regresiones lineales de los datos obtenidos en el resto de los años incluidos en el estudio.

Los datos del IDH se obtuvieron a partir de los informes del desarrollo humano del Programa de las Naciones Unidas para el Desarrollo para el intervalo de años comprendido entre 2010 y 2014 y para cada uno de los países participantes (31). Los datos del IDH correspondientes a 2015 se calcularon a través de regresiones lineales utilizando los datos de los 5 años anteriores. Esto se hizo con la finalidad de poder determinar si existe una asociación entre la prevalencia de DM2 y el IDH en el periodo evaluado. Se eliminaron aquellos países que carecían de información suficiente para calcular el IDH o la prevalencia de DM2.

Además, se analizaron las prevalencias de DM2 mayores y menores notificadas entre 2010 y 2015 y se calcularon las medias por país de las prevalencias de DM2 durante el mismo periodo sumando cada una de las prevalencias de DM2 y dividiendo entre 6. Lo anterior también se calculó para el IDH.

Para realizar el análisis estadístico se analizó correlación lineal entre la prevalencia de DM2 y el IDH con el coeficiente de correlación de Spearman para determinar su asociación y su dirección debido a que al analizar ambas variables las dos tuvieron una distribución no normal (*P* < 0,001). Se construyeron regresiones lineales entre ambas variables por país, región del mundo y continente. Los análisis estadísticos se llevaron a cabo con el paquete estadístico Stata v.12 fijando como nivel de significación estadística una alfa = 0,05 (32).

## RESULTADOS

De un total de 220 países de las siete regiones de la FID, se eliminaron 38 por falta de datos sobre la prevalencia de DM2 o del IDH, y se incluyeron, finalmente, 182 distribuidos de la siguiente manera: Oriente medio y Norte de África 10,4%, Europa 28,6%, África 23,6%, América del Norte y el Caribe 9,3%, América Central y del Sur 9,9%, Pacífico Occidental 14,3%, y Sudeste asiático 3,8%. (cuadro 1).

La prevalencia de DM2 entre 2010 y 2015 varió entre 0,8% (Benín, África) y 37,3% (Micronesia, Pacífico Occidental). Los países que notificaron menor prevalencia de DM2 (entre 0,8 y 2,2%) durante los seis años analizados fueron Benín, Malí, Ruanda, Mongolia, Islandia, Burundi, Gambia, Uganda, Burkina Faso, Guinea, Guinea Bissau, Níger, Senegal y Sierra Leona, de los cuales 85,7% pertenecían a África. Los países con mayor prevalencia de DM2 (entre 20,0 y 37,3%) en el mismo periodo fueron Micronesia, Kiribati, Arabia Saudita, Baréin, Kuwait, Qatar, Vanuatu, Mauricio, Líbano y Palau, de los cuales 50,0% pertenecían a Oriente Medio y Norte de África.

Según las medias de las prevalencias calculadas por país, aquellos con menor prevalencia fueron Benín, Malí, Gambia, Burundi, Mozambique, Burkina Faso, Guinea-Bissau, Sierra Leona, Uganda y Camboya, de los cuales 90,0% pertenecían a la región de África. Por otro lado, los países con la media de prevalencia más alta fueron Egipto, Mauricio, Vanuatu, Emiratos árabes Unidos, Baréin, Qatar, Kuwait, Arabia Saudita, Kiribati y Micronesia, 6,0% de los cuales pertenecían a la región del Oriente Medio y África, y 30,0%, al sudeste asiático. La región con las medias más elevadas de la prevalencia de DM2 en el período estudiado fue Oriente Medio y Norte de África. Las medias de las prevalencias de los países de esta región fueron diez puntos porcentuales mayores durante los últimos años evaluados y lo opuesto ocurrió en la región de África, donde se registraron las menores medias de prevalencia de DM2 (cuadro 2).

El IDH en los últimos seis años varió entre 0,3256 (Níger, África) y 0,9444 (Noruega, Europa). Los países con menor IDH (0,3256–0,3999) en el mismo periodo fueron Níger, Chad, Burkina Faso, República Centroafricana, Eritrea, Sierra Leona, Guinea y Burundi, todos pertenecientes a África, y aquellos con mayor IDH (0,915– 0,9444) entre 2010 y 2015, Noruega, Australia, Suiza, Dinamarca, Países Bajos, Alemania, Irlanda, Canadá, Estados Unidos de América y Singapur, 63,3% de los cuales pertenecían a Europa.

En cuanto a las medias del IDH por país en el mismo periodo, aquellos con la media más baja (IDH < 0,416) fueron Níger, República Centroafricana, Chad, Eritrea, Burkina Faso, Burundi, Sierra Leona, Guinea, Mozambique y Mali, todos pertenecientes a la región de África. Por su parte, los países con la media más alta fueron Nueva Zelanda, Canadá, Irlanda, Estados Unidos de América, Alemania, Países Bajos, Dinamarca, Suiza, Australia y Noruega, (IDH > 0,91). Europa fue la región con mayor IDH y África con el menor (cuadro 2).

Al estimar la prevalencia de DM2 según el IDH se observó que los países con mayor IDH tenían una mayor prevalencia de DM2 y viceversa (*P* < 0,001). En los países con IDH menor de 0,60 la prevalencia de DM2 fue menor de 7 (cuadro 3).

**FIGURA 1.**
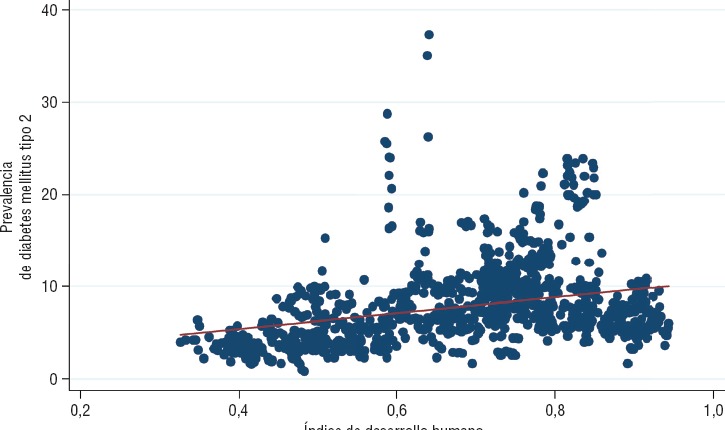
Correlación entre la prevalencia de diabetes mellitus tipo 2 (DM2) y el índice de desarrollo humano (IDH)

 El coeficiente de correlación de Spearman entre el IDH y la prevalencia de DM2 fue de 0,339 (*P* < 0,001) (figura 1). Aunque es una asociación leve-moderada, puede observarse una tendencia según la cual a mayor IDH, más alta es la prevalencia de DM2. En los modelos de regresión se observó que la relación entre la variable epidemiológica y la social fue estadísticamente significativa e inversa en el conjunto de los países y años incluidos (r2 = 9,11, constante = 1,9, coeficiente de regresión = 8,6, *P* < 0,001). Al analizar esta asociación por años, esta asociación fue estadísticamente significativa y con el mismo patrón en cada uno de los años estudiados (*P* < 0,05) (cuadro 4).

 Teniendo en cuenta las regiones de la FID, se encontró que la asociación entre el IDH y la prevalencia de DM2 fue estadísticamente significativa y con el mismo patrón para las regiones de África, Oriente Medio y Norte de África y en el Sudeste asiático, y que el modelo de regresión con el IDH como variable independiente explica hasta 50,9% de la varianza de la prevalencia de la DM2 (*P* < 0,001) (cuadro 4).

**CUADRO 1.** Número de países incluidos en el estudio según la Federación Internacional de Diabetes (FID) entre 2010 y 2015

Región de la FID	Países pertenecientes a la FID	Países eliminados	Total de países incluidos	Porcentaje de inclusión
Oriente Medio y Norte de África	21	2	19	90,48
Europa	56	4	52	92,86
África	49	6	43	87,76
América del Norte y el Caribe	28	11	17	60,71
América Central y del Sur	20	2	18	90,00
Pacífico Occidental	39	13	26	66,67
Sudeste asiático	7	0	7	100
Total	220	38	182	82,73

***Fuente:*** elaboración propia a partir de la información obtenida de la referencia 30.

**CUADRO 2.** Medias de la prevalencia de diabetes mellitus tipo 2 (DM2) y del índice desarrollo humano (IDH) por región de la Federación Internacional de Diabetes de 2010 a 2015

Región		Medias de la prevalencia de DM2				
2010^a^	2011^b^	2012^c^	2013^d^	2014^e^	2015^f^
África	4,2	4,8	4,7	5,3	4,6	4,2
Europa	6,6	6,2	6,1	6,0	6,6	7,0
Sudeste asiático	8,1	8,8	8,1	7,6	8,5	10,1
América Central y del Sur	7,5	8,5	8,4	8,1	8,8	9,1
Pacífico Occidental	6,6	9,4	10,3	10,6	10,5	10,2
América del Norte y el Caribe	9,6	11,5	12,1	11,3	12,0	12,1
Oriente Medio y Norte de África	10,8	13,5	13,6	13,6	13,3	12,7
A nivel mundial	6,9	7,9	8,0	8,0	8,2	8,2
Región		Medias del IDH				
2010^g^	2011^g^	2012^g^	2013^g^	2014^g^	2015^h^
África	0,4823	0,4887	0,4938	0,4975	0,5012	0,5067
Europa	0,8137	0,8178	0,8208	0,8231	0,8251	0,8286
Sudeste asiático	0,6303	0,6384	0,6439	0,6483	0,6530	0,6595
América Central y del Sur	0,7182	0,7170	0,7211	0,7236	0,7258	0,7298
Pacífico Occidental	0,7111	0,7152	0,7185	0,7209	0,7233	0,7184
América del Norte y el Caribe	0,7378	0,7398	0,7417	0,7423	0,7445	0,7460
Oriente Medio y Norte de África	0,6965	0,6963	0,7015	0,7023	0,7020	0,7049
A nivel mundial	0,6844	0,6886	0,6924	0,6949	0,6972	0,6999

***Fuente:*** elaboración propia a partir del análisis de la información de las bases de datos de los países seleccionados (referencias 26–30). http://www.anlis.gov.ar/cenagem/?page_id=584 y de Costa Rica: https://www.inciensa.sa.cr/actualidad/Informes%20de%20vigilancia.aspx#HERMES_TABS_1_5

a Federación Internacional de Diabetes. Diabetes atlas de la FID. 4ta ed. FID 2010.

b Federación Internacional de Diabetes.Diabetes atlas de la FID. 5ta ed. FID 2011.

c Federación Internacional de Diabetes. Diabetes atlas de la FID. 5ta ed. FID. Actualización 2012.

d Federación Internacional de Diabetes. Diabetes atlas de la FID. 6ta ed. FID 2013.

e Prevalencia calculada a partir de los años 2010, 2011, 2012, 2012, 2015.

f Federación Internacional de Diabetes. Diabetes atlas de la FID. 7ma ed. FID 2015. (referencias 26–30).

g Referencia 31.

h Datos calculados a partir de los 5 años anteriores.

## DISCUSIÓN

Los estudios ecológicos tienen limitaciones que pueden afectar y explicar los resultados obtenidos. Uno de los principales problemas está relacionado con la calidad de los datos, ya que los sistemas de información en salud de los diferentes países son muy heterogéneos como consecuencia de problemas relacionados con su registro asistemático, la ausencia de soporte magnético, la carencia de formalización institucional de las bases de datos institucional o superposiciones e incoherencias entre bases de datos semejantes, que podrían ocasionar un sobre o subregistro tanto de la prevalencia de DM2 como de la mortalidad utilizada para calcular el IDH (33). Otro problema está relacionado con las pocas actividades de tamizaje y diagnóstico de la DM2 realizadas en diversos países, lo que podría desembocar en una subestimación de su prevalencia (34). Por último, en este tipo de estudios se toma como unidad de análisis grupos poblacionales y no al individuo, por lo que la extrapolación de resultados debe hacerse con cautela (33).

**CUADRO 3.** Medias de la prevalencia de diabetes mellitus tipo 2 (DM2) según el índice de desarrollo humano (IDH)

IDH	n	Prevalencia de DM2		
		Media	Mínima	Máxima
0,3000–0,3999	37	3,9	1,8	6,3
0,4000–0,4999	140	4,4	0,8	9,9
0,5000–0,5999	133	6,6	2,3	27,8
0,6000–0,6999	157	8,5	1,6	37,3
0,7000–0,7999	332	9,7	2,5	22,3
0,8000–0,8999	201	8,9	1,6	23,9
0,9000–0,9999	92	6,7	3,2	10,7
P	<0,001	

***Fuente:*** elaboración propia a partir del análisis de la información de las bases de datos de los países seleccionados (referencias 26–31).

***Fuente:*** elaboración propia a partir del análisis de la información de las bases de datos de los países seleccionados (referencias 26–31).

**CUADRO 4.** Regresiones lineales para la prevalencia de DM2 según la variación del índice de desarrollo humano por año y región de la FID entre 2010 y 2015

	Constante	Coeficiente	R^2^	P
2010	0,5	9,4	0,224	<0,001
2011	2,3	8,2	0,092	<0,001
2012	2,0	8,7	0,075	<0,001
2013	3,9	6,0	0,036	0,0106
2014	2,2	8,6	0,086	<0,001
2015	1,1	10,1	0,126	<0,001
2010–2015	1,9	8,6	0,0911	<0,001
África	-3,1	15,5	0,3734	<0,0001
América Central y del Sur	12,3	-5,5	0,0362	0,0487
América del Norte y el Caribe	10,1	1,8	0,0043	0,5134
Europa	7,8	-1,7	0,0050	0,2127
Oriente Medio y Norte de África	-8,4	30,4	0,5091	<0,0001
Pacífico Occidental	11,4	-2,50	0,0032	0,4851
Sudeste asiático	-12,0	31,9	0,4614	<0,0001

***Fuente:***elaboración propia a partir del análisis de la información de las bases de datos de los países seleccionados (referencias 26–31).

No obstante lo anterior, estos estudios son sencillos, fáciles de realizar, de bajo costo y en ellos se utiliza un diseño rápido que permite averiguar la existencia de asociaciones entre fenómenos a nivel poblacional, y en este caso, entre un determinante social y un problema de salud pública mundial. Además, son útiles para formular nuevas hipótesis, sobre todo a partir de la información que se extrae de bases de datos (35).

En el presente estudio se encontró que 9% de la prevalencia global de DM2 está explicada por el IDH. Sin embargo, hubo regiones, como Oriente Medio y Norte de África, donde este valor alcanzó 50% y donde se estimaron las medias de prevalencia de DM2 más elevadas. Estos hallazgos indican que un mayor nivel de vida se traduce en mayores prevalencias de DM2. Además, aunque el coeficiente de correlación de Spearman entre el IDH y la prevalencia de DM2 fuese 0,339 y estadísticamente significativo, la correlación es sólo leve-moderada.

Los resultados antes mencionados son incongruentes con los notificados en otros estudios publicados según los cuales a mayor IDH, menor inactividad física en países de bajos y medianos ingresos, porque la inactividad física es un factor de riesgo de la aparición de DM2 (36). Sin embargo,existen otros factores como el estrés y la dieta hipercalórica que desencadenan problemas como la obesidad, el principal factor de riesgo para desarrollar DM2 (37, 38).

Por otro lado, en otros estudios donde se buscó una asociación entre el IDH y la prevalencia de depresión (39) y tuberculosis (10) se comprobó que en los países con menor IDH la prevalencia de estos padecimientos fue la más alta, unos resultados que son opuestos a lo observado en la presente investigación probablemente porque pertenecer a un nivel socioeconómico alto implica otros factores relacionados con el estrés al que se somete una persona a diario, como la presión que ejercen cierto tipos de trabajo. En cambio, cuando se intentó asociar el IDH con problemas de salud mental, no se encontró una tendencia lineal, lo que confirma un comportamiento errático de las asociaciones con el IDH (36).

En conclusión, a pesar de que el IDH incluye indicadores de salud, educación y el PIB, la relación de este indicador con la prevalencia de DM2 no muestra una tendencia lineal clara debido a que la DM2 es una enfermedad multifactorial y a que, además, podrían influir factores propios del diseño del estudio. Por lo tanto, se propone que, a partir de la información obtenida, se realicen más investigaciones con otro tipo de diseño, que incluya determinantes sociales y permita conocer cómo se generan los mecanismos de protección y de riesgo de la DM2 para poder diseñar estrategias específicas adaptadas a cada región o contexto para mejorar la salud de la población.

### Financiación.

Este estudio no recibió ningún tipo de financiación.

### Declaración.

Las opiniones expresadas por los autores son de su exclusiva responsabilidad y no reflejan necesariamente los criterios ni la política de la *RPSP/PAJPH* o de la OPS.
